# The Promise of Targeting Hypoxia to Improve Cancer Immunotherapy: Mirage or Reality?

**DOI:** 10.3389/fimmu.2022.880810

**Published:** 2022-06-20

**Authors:** Bassam Janji, Salem Chouaib

**Affiliations:** ^1^ Tumor Immunotherapy and Microenvironment (TIME) group, Department of Cancer Research. Luxembourg Institute of Health (LIH), Luxembourg City, Luxembourg; ^2^ Institut National de la Santé et de la Recherche Médicale (INSERM) Unités Mixtes de Recherche (UMR) 1186, Integrative Tumor Immunology and Genetic Oncology, Gustave Roussy, Villejuif, France; ^3^ Thumbay Research Institute of Precision Medicine, Gulf Medical University, Ajman, United Arab Emirates

**Keywords:** hypoxia, immune checkpoints, immune landscape, innate and adaptive immune response, pro-inflammatory chemokines, cancer immunotherapy, cold and hot tumor

## Abstract

Almost all solid tumors display hypoxic areas in the tumor microenvironment associated with therapeutic failure. It is now well established that the abnormal growth of malignant solid tumors exacerbates their susceptibility to hypoxia. Therefore, targeting hypoxia remains an attractive strategy to sensitize tumors to various therapies. Tumor cell adaptions to hypoxia are primarily mediated by hypoxia-inducible factor-1 alpha (HIF-1α). Sensing hypoxia by HIF-1α impairs the apoptotic potential of tumor cells, thus increasing their proliferative capacity and contributing to the development of a chaotic vasculature in the tumor microenvironment. Therefore, in addition to the negative impact of hypoxia on tumor response to chemo- and radio-therapies, hypoxia has also been described as a major hijacker of the tumor response by impairing the tumor cell susceptibility to immune cell killing. This review is not intended to provide a comprehensive overview of the work published by several groups on the multiple mechanisms by which hypoxia impairs the anti-tumor immunity and establishes the immunosuppressive tumor microenvironment. There are several excellent reviews highlighting the value of targeting hypoxia to improve the benefit of immunotherapy. Here, we first provide a brief overview of the mechanisms involved in the establishment of hypoxic stress in the tumor microenvironment. We then discuss our recently published data on how targeting hypoxia, by deleting a critical domain in HIF-1α, contributes to the improvement of the anti-tumor immune response. Our aim is to support the current dogma about the relevance of targeting hypoxia in cancer immunotherapy.

## Introduction

**Figure 1:**
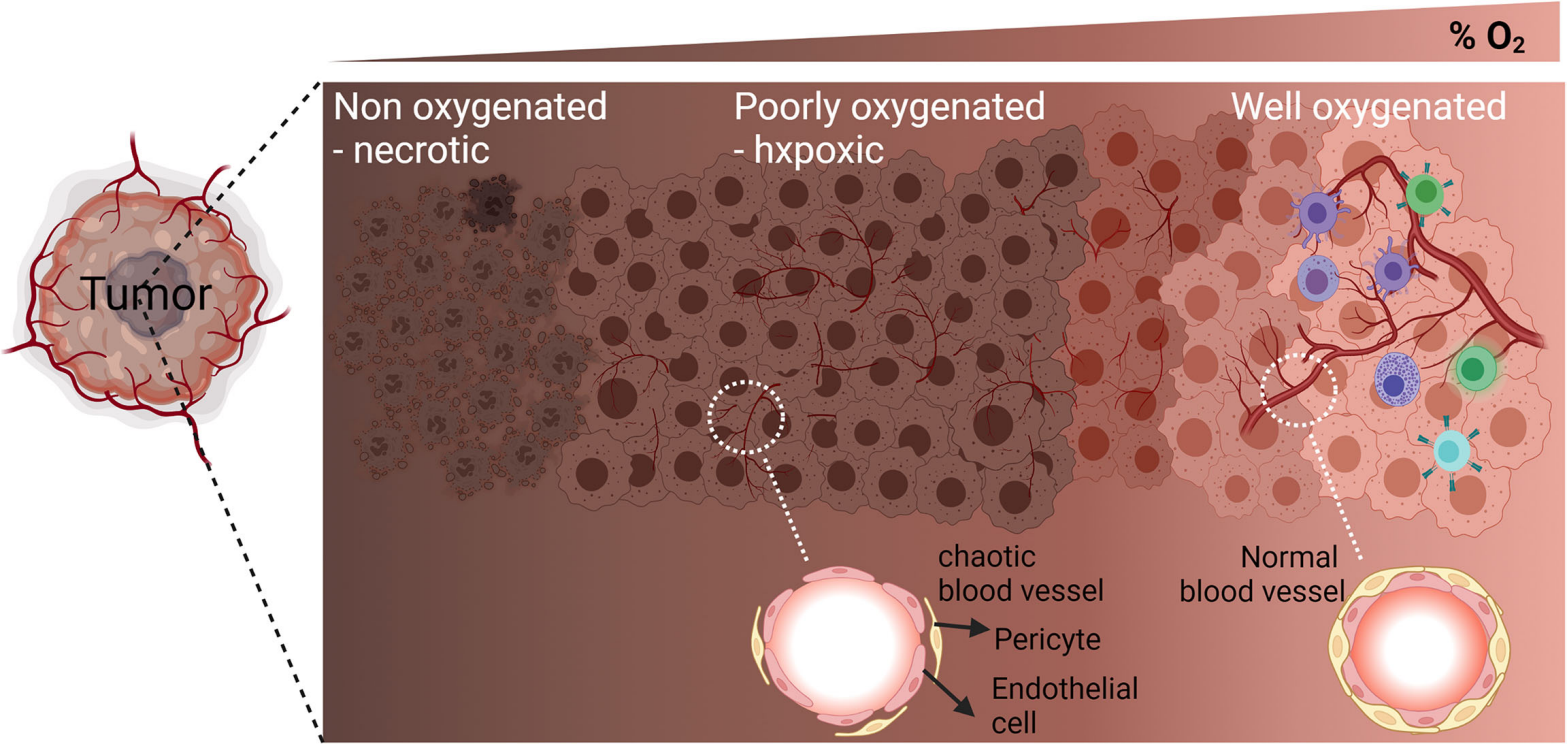
Graphic representation of the different areas in the tumor microenvironment according to the oxygenation level (percent of O_2_): Well oxygenated, poorly oxygenated, and non-oxygenated or necrotic areas. Enlargement of a blood vessel section in the poorly oxygenated hypoxic area shows defect in the organization of endothelial cells and pericytes’ coverage. Enlargement of a blood vessel section in the well oxygenated area shows well-structured endothelial cells and pericytes’ coverage.

**Figure 2:**
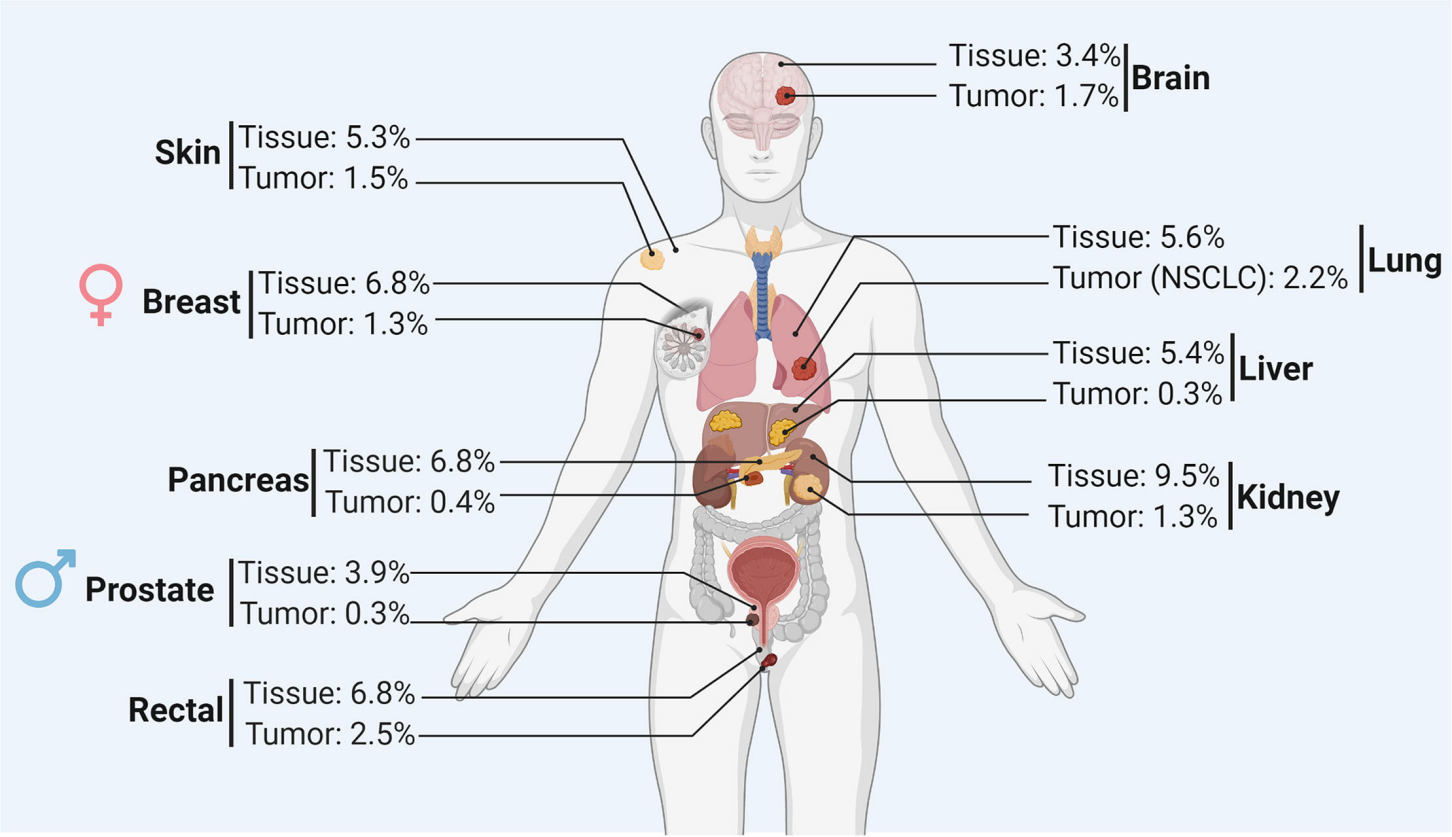
Summary of the oxygen level (reported as a percentage) in the healthy tissue and corresponding tumor of different organs.

In solid tumors, the establishment of hypoxia in the tumor microenvironment relies on the failure of abnormal vasculature to meet increasing oxygen demands from rapidly proliferating cancer cells. Therefore, within the same tumor, the O_2_ level varies depending on the quality and the integrity of blood vessels. Several areas in the tumor microenvironment can be identified according to the oxygenation level of tumor tissue: well oxygenated, poorly oxygenated, and non-oxygenated or necrotic areas (1) **(** **Figure 1** **)**. In addition to the tumor size and the quality of the tumor vascularization, the different levels of O_2_ in the microenvironment of different tumors rely on the initial physiological oxygenation levels observed in the corresponding healthy tissue and on the degree of the tumor heterogeneity. **Figure 2** shows the oxygen levels (reported as a percentage) in several tumors and corresponding healthy tissues. The percentage of O_2_ in healthy tissues range from 9.5% (observed in kidney healthy tissue) to 3.5% (reported in healthy prostate tissue). Hence, the average of O_2_ in the healthy tissues reported in **Figure 2** is 5.9%. The oxygen levels in the corresponding tumors range from 2.5% (observed in rectal tumor) to 0.3% (reported in liver and prostate tumors). Therefore, the average of O_2_ in the tumors reported in **Figure 2** is 1.3%. Based on these values, most tumors exhibit median oxygen levels below 2%. The term of normoxia should not be used to describe the oxygenation level in healthy tissues, however, it can defines the O2 level in tissue culture flasks where the oxygenation is about 20-21%. The term of physioxia is more appropriate to describe the oxygenation status in healthy tissues as previously reported (2). Therefore, it is important to control the O_2_ in cell culture settings to mimic as far as possible the O2 levels found in healthy and tumor tissues.

The mechanism of cell adaptation to hypoxia is currently well described. William G. Kaelin Jr., Sir Peter J. Ratcliffe, and Gregg L. Semenza were awarded the Nobel Prize in Medicine 2019 in recognition of their seminal discovery on the molecular mechanisms and signaling pathways by which cells sense and adapt to hypoxia.

While the negative impact of hypoxia on tumor response to conventional chemo- and radiotherapy is now well recognized (3, 4), an accumulating new body of data highlights its involvement in tumor resistance to immunotherapy (5). Here, we describe recent evidence on how hypoxia plays a role as a culprit of immunotherapy failure. We will mainly discuss our recent experimental and preclinical evidence data showing that strategies targeting hypoxia can provide the basis for innovative combination therapies that may improve the immunotherapeutic efficacy. Hypoxia-inducible factors (HIFs) are essential transcription factors mediating cell adaptation to hypoxia, and thus we will first briefly describe how HIFs expression and stability are regulated under hypoxia in tumor cells.

## Hypoxia Inducible Factors - Mechanisms of Regulation and Stability

HIFs are heterodimer complexes consistent of an O_2_-inducible alpha subunit and constitutively expressed beta subunit (HIF-1β/ARNT). Three alpha subunits have been identified: HIF-1α, HIF-2α, and HIF-3α. The well-studied alpha subunit is HIF-1α and contains N-terminal basic-helix-loop-helix (bHLH) required for DNA interaction. There are also two Per-Arnt-Sim (PAS) domains (PASa and PASb) essential for heterodimerization with HIF-1β. Two oxygen-dependent degradation domains (ODDD) have been identified in the N-terminal (N-ODDD) and C-terminal (C-ODDD) parts of the protein in addition to two transactivation domains (TADs). One overlaps with the C-ODDD, and the second is found in the C-terminal part (6).

Under normoxic conditions, HIF-1α is continuously synthesized, but it is rapidly degraded by the ubiquitin–proteasome system (UPS). The short half-life of HIF-1α under normoxia is less than five minutes (7). The basal expression level of HIF-1α under normoxia is low, but varies in different cells. Such variations depend on the rate of HIF-1α synthesis (O_2_-independent mechanism) and the rate of HIF-1α degradation (O_2_-dependent mechanism).

The degradation of HIF-1α under normoxia depends on its hydroxylation on proline residues located at positions 402 and/or 564 in the ODDD by prolyl hydroxylase domain protein 2 (PHD2). Thus, hydroxylated HIF-1α binds to von Hippel-Lindau (pVHL) protein, which is part of the E3 ubiquitin-protein ligase complex. It is subsequently subjected to degradation by the UPS [reviewed in (8)].

The enzymatic activity of PHD2 requires O_2_ as a substrate, and thus the protein becomes inactive in hypoxic cells (9). Therefore, HIF-1α is no longer hydroxylated under low O_2_ pressure; as a result, its interaction with pVHL and subsequent degradation by UPS are blocked. Thus, the failure of the mechanism involved in HIF-1α degradation under hypoxia leads to its accumulation in the cytoplasm, translocation to the nucleus, and interaction with HIF-1β. The heterodimer HIF-1α/HIF-1β binds to the hypoxia-responsive element (HRE) motif found in the promoter of several genes involved in several biological processes that tolerate cellular adaptation to hypoxia and confer a survival benefit to tumor cells.

HIF-2α displays similar DNA binding and dimerization domains as HIF-1α, but these differs in the transactivation domains (10). Therefore, the hydroxylation of HIF-2α is also regulated in an oxygen-dependent manner (11). Both HIF-1α and HIF-2α regulate common downstream target genes, but each can also regulate specific genes (12). Unlike HIF-1α and HIF-2α, HIF-3α lacks the transactivation domain. It can inhibit the activity of HIF-1α and HIF-2α (13), and HIFs are involved in the regulation of several microRNAs (HRM) (14) and chromatin-modifying enzymes (15). HIFs can directly regulate more than 800 genes involved in several biological functions as revealed by ChIP-seq analysis and genome-wide chromatin immunoprecipitation combined with DNA microarrays (ChIP-on-chip) (16, 17). The expression of downstream target genes is achieved by binding HIF-1α to 50-base pair cis-acting hypoxia responsive element (HRE) motifs found in their enhancer and promoter regions (18). The HRE motif contains the core sequence 5’-[A/G]CGT-3’, which is usually ACGTG (19). Considering the preferential binding of the heterodimer complex HIF-1α/HIF-1β to specific bases in the 5’ and 3’ ends of the HRE motif, the following HRE consensus sequence [T/G/C][A/G]**CGTG**[CGA][GTC][GTC][CTG] has been described (19).

## Strategies for Targeting Hypoxia - Challenges and Opportunities

Inhibiting hypoxia has inspired significant interest because it can improve therapeutic outcomes. Strategies used to inhibit hypoxia rely on bio-reductive prodrugs (20) or inhibitors targeting pathways upon which the survival of hypoxic cells depends (21). However, targeting HIF-dependent pathways is extremely challenging because various signaling pathways converge on—and emerge from—HIFs (22). Additional approaches have been proposed consisting of targeting HIFs directly. Although considerable efforts have been undertaken to identify selective inhibitors of HIFs, enthusiasm has been tempered by the reality that transcription factors, including HIFs, seem to be “undruggable” or at least no selective drugs inhibiting HIFs have been identified.

Considering the well-described molecular mechanism of HIF-1α protein activity, various strategies have been proposed to impair such activity. Such mechanisms inhibit HIF-1α protein synthesis or stabilization; they can also prevent HIF-1α/β heterodimerization or HIFs/DNA binding (23).

## Inhibiting Hypoxia by Preventing HIF-1α/β, Heterodimerization Regulates Pro-Inflammatory Chemokines and Improves the Benefit of Immunotherapies

**Figure 3:**
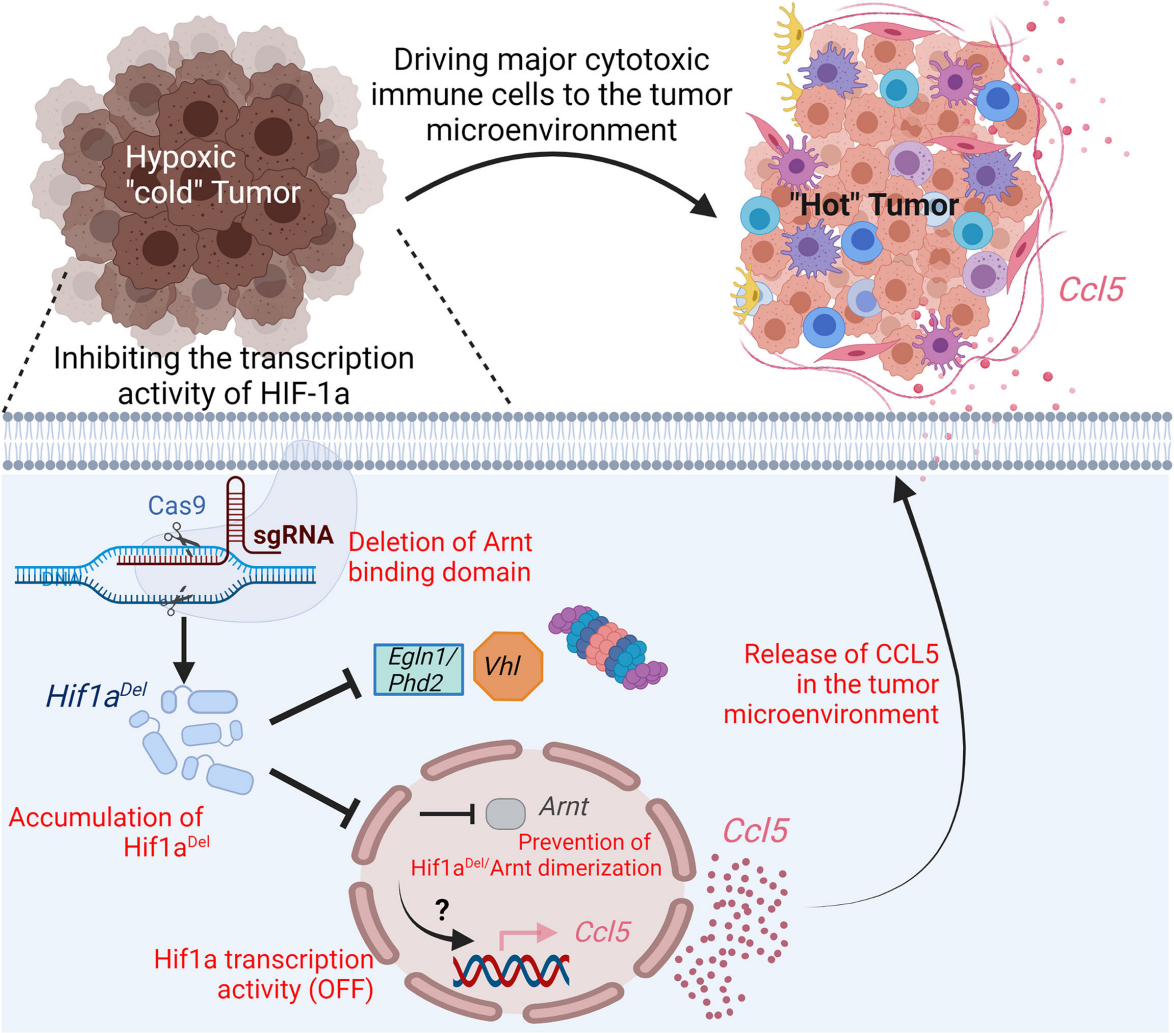
Impact of targeting the transcription activity of Hif1a on driving immune cells into melanoma tumor microenvironment. Hypoxic melanoma are “cold” poorly infiltrated by immune cells. Deletion, in Hif1a, of the domain responsible for the formation of a heterodimer with Arnt by CRISPR/Cas9 gene-editing technology, prevents its transcription activity. In hypoxic cells expressing deleted Hif1a^Del^, the pro-inflammatory (C-C motif) ligand 5 chemokine (Ccl5) is overexpressed by a mechanism which is not fully understood. The release of Ccl5 by tumor cells in the tumor microenvironment drives major cytotoxic immune cells and contributes to the establishment of pro-inflammatory “hot” tumor.

In a highly hypoxic and PD-1-resistant B16-F10 melanoma mouse model (24, 25), we recently reported that inhibiting hypoxia by preventing HIF-1α/β heterodimerization in a mouse melanoma model drives immune cells into the tumor microenvironment and improves anti-PD-1- and vaccine-based immunotherapies (26). Using CRISPR/Cas9 technology, we showed that the deletion (in HIF-1α) of the domain responsible for the interaction with HIF-1β still leads to the accumulation of the protein in hypoxic cells; however, this remarkably inhibits its transcription activity as demonstrated by suppressing the expression of well-known HIF-1α downstream target genes CAIX, VEGF, and Glut1. Similar to the full-length HIF-1α (HIF-1α^FL^), the deleted HIF-1α (hereafter reported to as HIF-1α^Del^) accumulated in the cytoplasm of hypoxic cells. However, unlike HIF-1α^FL^, HIF-1α^Del^ displayed a defect in the nuclear translocation as seen *via* confocal microscopy analysis. By assessing the tumor growth *in vivo*, we showed a significant decrease in the growth and weight of B16-F10 tumors expressing HIF-1α^Del^ versus those expressing HIF-1α^FL^. Such effects were observed in immunocompetent but not in immunocompromised NOD scid gamma (NSG) mice lacking mature B, T, and NK cells (26). These data emphasize that targeting hypoxia in tumors inhibits tumor growth *via* the immune system. Indeed, we revealed a significant increase in the infiltration of CD45+, NK, CD4+, and CD8+ cells into HIF-1α^Del^ versus HIF-1α^FL^ **(** **Figure 3** **)**. These data strongly suggest that targeting the transcription activity of HIFs can switch the microenvironment of tumors from cold non-inflamed/not-infiltrated into hot inflamed and infiltrated by cytotoxic immune cells.

The infiltration and trafficking of immune cells to the tumor microenvironment relies on the establishment of a chemokine network. The recruitment of T cells and natural killer (NK) cells into the tumor can be achieved by chemokines CXCL9, 10, 11, 16 as well as CX3CL1. CCL19 and 21 can promote the recruitment of DCs into T-cell priming sites, thus leading to T-cell activation (27). CXCL16 has been associated with the infiltration of tumor-infiltrating lymphocytes (TILs) and better prognosis in colorectal cancer (28). We previously reported that driving NK cells to melanoma tumors depends on the release of CCL5 to the tumor microenvironment by tumor cells (29). Other studies showed that the chemokines CCL2, 3, 4, and 5 as well as CXCL9 and 10 were involved in T-cell migration into a melanoma tumor microenvironment (30). By assessing the chemokine network in HIF-1α^Del^ tumors, we see that the increased infiltration of major cytotoxic immune cells described above was associated with the release of proinflammatory chemokines in the tumor microenvironment—notably CCL5 and CCL2. Therefore, we believe that targeting the transcriptional activity of HIF-1α in tumor cells contributes to the establishment of an inflammatory microenvironment, which helps recruit cytotoxic immune effector cells.

The translational value of our study is underlined by the data generated in preclinical mouse model and using a cohort of melanoma patients. Treatment of melanoma-bearing mice with acriflavine, reported to prevent HIF-1α/HIF-1β heterodimerization, improved immunotherapy strategies based on TRP-2 peptide vaccination and anti-PD-1 antibody. We further showed that melanoma patients having low Winter hypoxia score survive better and show increased CCL5 as well as high tumor infiltration by NK and CD8 T-cells versus those having a high hypoxia score.

## HIF-1α Induces Tumor Escape From Immune Surveillance by Upregulating the Expression of Immune Checkpoints and Activating Various Survival Pathways in Tumor Cells

**Figure 4:**
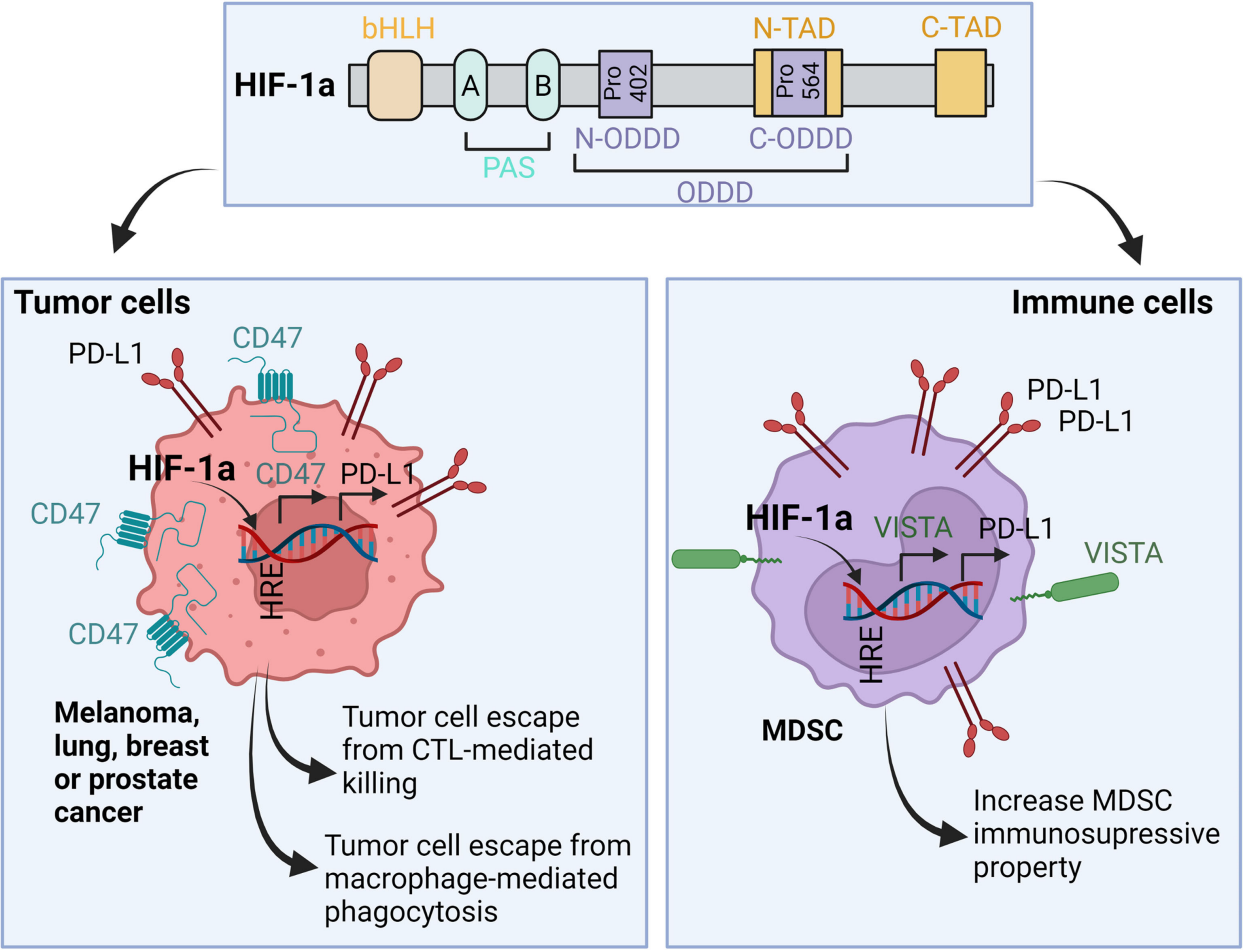
Role of HIF-1α in the regulation of immune checkpoints expression in both tumor and immune cells. In hypoxic microenvironment, HIF-1α binds to the HRE motifs found in the promoters of PD-L1, CD47 and VISTA. As a result, HIF-1α-depenedent overexpression of PD-L1 and CD47 in tumor cells leads to tumor escape from CTL-mediated killing and macrophage-mediated phagocytosis, respectively. In MDSC, HIF-1α-dependent upregulation of PD-L1 and VISTA increases their immunosuppressive properties in the tumor microenvironment. bHLH, basic-helix-loop-helix; PAS, Per-Arnt-Sim domains; Pro, Proline residue; N- and C-ODDD, NH_2_-terminal and COOH-terminal Oxygen-Dependent Degradation Domains; N- and C-TAD, NH_2_-terminal and COOH-terminal transactivation domain.

Accumulating evidence points to a critical role of HIFs in regulating various immune checkpoints [reviewed in (31)]. Briefly, HIF-1α binds directly to the HRE motif in the promoter of PD-L1 gene and induces its expression in various cancer cells such as melanoma, lung, breast, and prostate cancer. Such overexpression resulted in tumor escape from immune surveillance (32, 33) **(** **Figure 4** **)**. Similarly, the constitutive accumulation of HIF-2α in clear cell renal cell carcinoma (ccRCC), due to the mutation status of VHL, facilitates PD-L1 upregulation (34). In addition to tumor cells, HIF-1α also operates in the immune suppressive cells present in hypoxic tumor microenvironment. In MDSCs, HIF-1α directly upregulates PD-L1 expression resulting in impaired cytotoxic T lymphocytes (CTL) activity (32).

VISTA is an additional immune checkpoint regulated by HIF-1α. VISTA is expressed on several myeloid cells infiltrating hypoxic tumors including CD11b^high^Gr1+ MDSCs. The recruitment of MDSCs to the tumor microenvironment is mediated by hypoxia-dependent upregulation of stromal-derived factor 1 (SDF1, CXCL12) (35). HIF-1α, but not HIF-2α, binds to VISTA and induces its expression—this process in turn suppresses T-cell proliferation and activity (36) **(** **Figure 4** **).**

CD47 is an inhibitory immune checkpoint expressed on the cell surface of tumor cells and involved in blocking the phagocytosis following the interaction with its ligands: signal regulatory protein α (SIRPα) and thrombospondin-1 (TSP-1). These two proteins are expressed on the surface of macrophages and dendritic cells (37). CD47/SIRPa or TSP-1 interaction delivers a strong “don’t eat me” signal to block phagocytosis (38). Upregulation of CD47 is associated with the expression of HIF-1α downstream target genes. The expression of CD47 is upregulated by HIF-1α in triple-negative breast cancer cells resulting in a stem cell phenotypic switch through which cancer cells escape from phagocytosis (39). The upregulation of CD47 by hypoxia has also been reported in pancreatic adenocarcinoma (40, 41) **(** **Figure 4** **).**

In addition to regulating the expression of immune checkpoints and the establishing immunosuppressive tumor microenvironment, the accumulation of HIF-1α in tumor cells decreases tumor cell susceptibility to CTL-mediated lysis through several mechanisms [reviewed in (31)]. Briefly, these mechanisms include the activation of autophagy (24, 42), the upregulation of stem cell self-renewal transcription factor Nanog (43, 44), and the induction of microRNA (miR)-210 involved in repressing the non-receptor protein tyrosine phosphatase type 1 (PTPN1), homeobox A1 (HOXA1), and tumor protein p53-inducible protein 11 (TP53I11) (45).

Hypoxia also impairs NK-mediated killing of tumor cells by downregulating and/or shedding the major histocompatibility complex (MHC) class I polypeptide-related sequence A (MICA) on the surface of cancer cells (46, 47). In hypoxic tumor cells, the activation of autophagy leads to the degradation of the serine protease granzyme B (GZMB) released by NK cells. This in turn led to tumor escape from NK-mediated killing (48, 49).

In addition of NK cells, hypoxia also impacts the activity of T cells. Briefly, under hypoxia, activated T cells are able to adapt changes in energy supplies by switching their metabolism to glycolysis and regulating extracellular-adenosine receptor signaling. Such adaptation alter the balance between T helper 1 cells and T helper 2 cells and results in impairing the anti-tumor immune response [reviewed in (50)]. In this context, it should be highlighted that hypoxia-dependent regulation of A2A adenosine receptor (A2AR)–mediated signaling is considered as one of the major mechanisms of the establishment of immunosuppressive tumor microenvironment [reviewed in (51)]

## Targeting Hypoxia: A Tricky Approach

Several reports indicate that the increased tumor aggressiveness is partially associated from hypoxia-induced genomic instability. It is currently well established that tumor cells exposed to hypoxic stress are able to acquire genetic instability through altered translation of DNA repair proteins. Therefore, hypoxic tumor cells display defective repair as well as an increased mutation rate. It is widely admitted that PD-L1 expression, tumor mutation burden (TMB) development, immune cell infiltration at the tumor site and neoantigen load are all thought to be influenced by tumor genomic instability (52). Clearly a more holistic approach that considers the complexity of hypoxia effects to better discriminate between the beneficial roles of hypoxic stress from the hostile ones is crucial. Given the dual effect of hypoxia, a clear understanding of how hypoxic stress induces tumor resistance and genomic instability resulting in an increased tumor immunogenicity is of paramount importance for identifying the time window of hypoxia targeting to improve cancer immunotherapy. Nevertheless, there is currently a Phase III clinical trial (NCT04195750) aiming to compare the efficacy and safety of HIF-2α inhibitor MK-6482 (also known as WELIREG) with the mTOR inhibitor everolimus in previously treated advanced ccRCC patients. Among the patients enrolled in the trial are those treated with anti–PD-1/PD-L1 or VEGF-targeted therapy which are randomly assigned to MK-6482 or everolimus arm. The estimated study completion will be in 2025. WELIREG or MK-6482 is the first inhibitor approved in U.S. which reduces the transcription and expression of HIF-2α target genes associated with cellular proliferation, angiogenesis and tumor growth.

## Concluding Remarks

This review provides an additional clue supporting the role of targeting hypoxia in improving the benefit of cancer immunotherapy. Hypoxia has long been considered an attractive target to overcome resistance and improve the benefits to various therapies including immunotherapy. Numerous strategies have been proposed to inhibit hypoxia and target the transcription activity of HIF-1α such as the development of hypoxia-activated prodrugs or small molecules interfering with the transcription activity of HIFs (53–55). Several experimental studies offer preclinical proof-of-concept that strategies targeting hypoxia can improve the therapeutic benefits of current cancer therapies. However, there are still no approved drugs that selectively target hypoxia or HIF-dependent pathways despite they have clear anticancer effects. Obviously, such lack of selectivity does not disqualify these drugs as anticancer agents, but it becomes challenging to attribute the potential effect observed in patients to their anti-hypoxic properties. Nevertheless, the failure of developing selective drugs could be attributed to the biological complexity of HIF-1α pathways. Indeed, HIF-1α controls a highly complex network connecting several signaling pathways and various overlapping mechanisms in tumor cells and other cells in the tumor microenvironment. Such properties make HIF-1α undruggable. Therefore, we strongly believe that better dissecting hypoxia-inducible responses and understanding HIF-dependent signaling would lead to novel targets and new treatment opportunities.

The key role of hypoxia in hijacking the anti-tumor immune response is now firmly grounded in a substantial body of research. Therefore, the use of hypoxia modulators—especially those interfering with the transcription activity of HIF-1α—holds much promise for improving the therapeutic benefit of cancer immunotherapies. There is no doubt that combining hypoxia modulators with cancer immunotherapy approaches provide a unique opportunity for innovative combination strategies. Additional efforts are needed for highly selective hypoxia inhibitors, which remain an unmet need and are among the greatest challenges in cancer therapy.

## Author Contributions

BJ and SC contributed to writing the manuscript and preparing the figures. All authors contributed to the article and approved the submitted version.

## Funding

This work was supported by grants from Luxembourg National Research Fund (BRIDGES2020/BM/15412275/SMART COMBO and BRIDGES2021/BM/16358198/TRICK-ALDH INTER/EUROSTARS21/16896480/C2I), FNRS-Televie (7.4560.21 INCITE21, 7.4579.20 CD73 and 7.4559.2 IMPACT21), Roche Pharma, “Fondation Recherche Cancer et Sang”, Luxembourg (INCOM BIOM) and Sheik Hamdan Bin Rashid Al Maktoum Foundation, United Arab Emirates.

## Conflict of Interest

The authors declare that the research was conducted in the absence of any commercial or financial relationships that could be construed as a potential conflict of interest.

## Publisher’s Note

All claims expressed in this article are solely those of the authors and do not necessarily represent those of their affiliated organizations, or those of the publisher, the editors and the reviewers. Any product that may be evaluated in this article, or claim that may be made by its manufacturer, is not guaranteed or endorsed by the publisher.

## References

[1] Singleton DC Macann A Wilson WR. Therapeutic Targeting of the Hypoxic Tumour Microenvironment. Nat Rev Clin Oncol (2021) 18(12):751–72. doi: 10.1038/s41571-021-00539-4 34326502

[2] McKeown SR. Defining Normoxia, Physoxia and Hypoxia in Tumours-Implications for Treatment Response. Br J Radiol (2014) 87(1035):20130676. doi: 10.1259/bjr.20130676 24588669 PMC4064601

[3] McAleese CE Choudhury C Butcher NJ Minchin RF. Hypoxia-Mediated Drug Resistance in Breast Cancers. Cancer Lett (2021) 502:189–99. doi: 10.1016/j.canlet.2020.11.045 33278499

[4] Chedeville AL Madureira PA. The Role of Hypoxia in Glioblastoma Radiotherapy Resistance. Cancers (Basel) (2021) 13(3):542. doi: 10.3390/cancers13030542 33535436 PMC7867045

[5] Kopecka J Salaroglio IC Perez-Ruiz E Sarmento-Ribeiro AB Saponara S De Las Rivas J. Hypoxia as a Driver of Resistance to Immunotherapy. Drug Resist Update (2021) 59:100787. doi: 10.1016/j.drup.2021.100787 34840068

[6] Semenza GL. Evaluation of Hif-1 Inhibitors as Anticancer Agents. Drug Discovery Today (2007) 12(19-20):853–9. doi: 10.1016/j.drudis.2007.08.006 17933687

[7] Lisy K Peet DJ. Turn Me On: Regulating Hif Transcriptional Activity. Cell Death Differ (2008) 15(4):642–9. doi: 10.1038/sj.cdd.4402315 18202699

[8] Masoud GN Li W. Hif-1alpha Pathway: Role, Regulation and Intervention for Cancer Therapy. Acta Pharm Sin B (2015) 5(5):378–89. doi: 10.1016/j.apsb.2015.05.007 PMC4629436 26579469

[9] Yang M Su H Soga T Kranc KR Pollard PJ. Prolyl Hydroxylase Domain Enzymes: Important Regulators of Cancer Metabolism. Hypoxia (Auckl) (2014) 2:127–42. doi: 10.2147/HP.S47968 PMC5045062 27774472

[10] Infantino V Santarsiero A Convertini P Todisco S Iacobazzi V. Cancer Cell Metabolism in Hypoxia: Role of Hif-1 as Key Regulator and Therapeutic Target. Int J Mol Sci (2021) 22(11):5703. doi: 10.3390/ijms22115703 34071836 PMC8199012

[11] Patel SA Simon MC. Biology of Hypoxia-Inducible Factor-2alpha in Development and Disease. Cell Death Differ (2008) 15(4):628–34. doi: 10.1038/cdd.2008.17 PMC2882207 18259197

[12] Lau KW Tian YM Raval RR Ratcliffe PJ Pugh CW. Target Gene Selectivity of Hypoxia-Inducible Factor-Alpha in Renal Cancer Cells Is Conveyed by Post-DNA-Binding Mechanisms. Br J Cancer (2007) 96(8):1284–92. doi: 10.1038/sj.bjc.6603675 PMC2360163 17387348

[13] Albadari N Deng S Li W. The Transcriptional Factors Hif-1 and Hif-2 and Their Novel Inhibitors in Cancer Therapy. Expert Opin Drug Discov (2019) 14(7):667–82. doi: 10.1080/17460441.2019.1613370 PMC6559821 31070059

[14] Kulshreshtha R Davuluri RV Calin GA Ivan M. A Microrna Component of the Hypoxic Response. Cell Death Differ (2008) 15(4):667–71. doi: 10.1038/sj.cdd.4402310 18219318

[15] Wu MZ Tsai YP Yang MH Huang CH Chang SY Chang CC. Interplay Between Hdac3 and Wdr5 Is Essential for Hypoxia-Induced Epithelial-Mesenchymal Transition. Mol Cell (2011) 43(5):811–22. doi: 10.1016/j.molcel.2011.07.012 21884981

[16] Xia X Lemieux ME Li W Carroll JS Brown M Liu XS. Integrative Analysis of Hif Binding and Transactivation Reveals Its Role in Maintaining Histone Methylation Homeostasis. Proc Natl Acad Sci USA (2009) 106(11):4260–5. doi: 10.1073/pnas.0810067106 PMC2657396 19255431

[17] Schodel J Oikonomopoulos S Ragoussis J Pugh CW Ratcliffe PJ Mole DR. High-Resolution Genome-Wide Mapping of Hif-Binding Sites by Chip-Seq. Blood (2011) 117(23):e207–17. doi: 10.1182/blood-2010-10-314427 PMC3374576 21447827

[18] Semenza GL Nejfelt MK Chi SM Antonarakis SE. Hypoxia-Inducible Nuclear Factors Bind to an Enhancer Element Located 3’ to the Human Erythropoietin Gene. Proc Natl Acad Sci USA (1991) 88(13):5680–4. doi: 10.1073/pnas.88.13.5680 PMC51941 2062846

[19] Wenger RH Gassmann M. Oxygen(Es) and the Hypoxia-Inducible Factor-1. Biol Chem (1997) 378(7):609–16. 9278140

[20] Anduran E Dubois LJ Lambin P Winum JY. Hypoxia-Activated Prodrug Derivatives of Anti-Cancer Drugs: A Patent Review 2006 - 2021. Expert Opin Ther Pat (2022) 32(1):1–12. doi: 10.1080/13543776.2021.1954617 34241566

[21] Wilson WR Hay MP. Targeting Hypoxia in Cancer Therapy. Nat Rev Cancer (2011) 11(6):393–410. doi: 10.1038/nrc3064 21606941

[22] Ratcliffe P Koivunen P Myllyharju J Ragoussis J Bovee JV Batinic-Haberle I. Update on Hypoxia-Inducible Factors and Hydroxylases in Oxygen Regulatory Pathways: From Physiology to Therapeutics. Hypoxia (Auckl) (2017) 5:11–20. doi: 10.2147/HP.S127042 28352643 PMC5359007

[23] Xia Y Choi HK Lee K. Recent Advances in Hypoxia-Inducible Factor (Hif)-1 Inhibitors. Eur J Med Chem (2012) 49:24–40. doi: 10.1016/j.ejmech.2012.01.033 22305612

[24] Noman MZ Janji B Kaminska B Van Moer K Pierson S Przanowski P. Blocking Hypoxia-Induced Autophagy in Tumors Restores Cytotoxic T-Cell Activity and Promotes Regression. Cancer Res (2011) 71(18):5976–86. doi: 10.1158/0008-5472.CAN-11-1094 21810913

[25] Noman MZ Parpal S Van Moer K Xiao M Yu Y Viklund J. Inhibition of Vps34 Reprograms Cold Into Hot Inflamed Tumors and Improves Anti-Pd-1/Pd-L1 Immunotherapy. Sci Adv (2020) 6(18):eaax7881. doi: 10.1126/sciadv.aax7881 32494661 PMC7190323

[26] Lequeux A Noman MZ Xiao M Van Moer K Hasmim M Benoit A. Targeting Hif-1 Alpha Transcriptional Activity Drives Cytotoxic Immune Effector Cells Into Melanoma and Improves Combination Immunotherapy. Oncogene (2021) 40(28):4725–35. doi: 10.1038/s41388-021-01846-x PMC8282500 34155342

[27] Gorbachev AV Fairchild RL. Regulation of Chemokine Expression in the Tumor Microenvironment. Crit Rev Immunol (2014) 34(2):103–20. doi: 10.1615/critrevimmunol.2014010062 PMC7191635 24940911

[28] Hojo S Koizumi K Tsuneyama K Arita Y Cui Z Shinohara K. High-Level Expression of Chemokine Cxcl16 by Tumor Cells Correlates With a Good Prognosis and Increased Tumor-Infiltrating Lymphocytes in Colorectal Cancer. Cancer Res (2007) 67(10):4725–31. doi: 10.1158/0008-5472.CAN-06-3424 17510400

[29] Mgrditchian T Arakelian T Paggetti J Noman MZ Viry E Moussay E. Targeting Autophagy Inhibits Melanoma Growth by Enhancing Nk Cells Infiltration in a Ccl5-Dependent Manner. Proc Natl Acad Sci USA (2017) 114(44):E9271–E9. doi: 10.1073/pnas.1703921114 PMC5676879 29078276

[30] Harlin H Meng Y Peterson AC Zha Y Tretiakova M Slingluff C. Chemokine Expression in Melanoma Metastases Associated With Cd8+ T-Cell Recruitment. Cancer Res (2009) 69(7):3077–85. doi: 10.1158/0008-5472.CAN-08-2281 PMC3886718 19293190

[31] Noman MZ Hasmim M Lequeux A Xiao M Duhem C Chouaib S. Improving Cancer Immunotherapy by Targeting the Hypoxic Tumor Microenvironment: New Opportunities and Challenges. Cells (2019) 8(9):1083. doi: 10.3390/cells8091083 PMC6770817 31540045

[32] Noman MZ Desantis G Janji B Hasmim M Karray S Dessen P. Pd-L1 Is a Novel Direct Target of Hif-1alpha, and Its Blockade Under Hypoxia Enhanced Mdsc-Mediated T Cell Activation. J Exp Med (2014) 211(5):781–90. doi: 10.1084/jem.20131916 PMC4010891 24778419

[33] Barsoum IB Smallwood CA Siemens DR Graham CH. A Mechanism of Hypoxia-Mediated Escape From Adaptive Immunity in Cancer Cells. Cancer Res (2014) 74(3):665–74. doi: 10.1158/0008-5472.CAN-13-0992 24336068

[34] Messai Y Gad S Noman MZ Le Teuff G Couve S Janji B. Renal Cell Carcinoma Programmed Death-Ligand 1, a New Direct Target of Hypoxia-Inducible Factor-2 Alpha, Is Regulated by Von Hippel-Lindau Gene Mutation Status. Eur Urol (2016) 70(4):623–32. doi: 10.1016/j.eururo.2015.11.029 26707870

[35] Zou W Chen L. Inhibitory B7-Family Molecules in the Tumour Microenvironment. Nat Rev Immunol (2008) 8(6):467–77. doi: 10.1038/nri2326 18500231

[36] Deng J Li J Sarde A Lines JL Lee YC Qian DC. Hypoxia-Induced Vista Promotes the Suppressive Function of Myeloid-Derived Suppressor Cells in the Tumor Microenvironment. Cancer Immunol Res (2019) 7(7):1079–90. doi: 10.1158/2326-6066.CIR-18-0507 PMC6606337 31088847

[37] Jaiswal S Jamieson CH Pang WW Park CY Chao MP Majeti R. Cd47 Is Upregulated on Circulating Hematopoietic Stem Cells and Leukemia Cells to Avoid Phagocytosis. Cell (2009) 138(2):271–85. doi: 10.1016/j.cell.2009.05.046 PMC2775564 19632178

[38] Willingham SB Volkmer JP Gentles AJ Sahoo D Dalerba P Mitra SS. The Cd47-Signal Regulatory Protein Alpha (Sirpa) Interaction Is a Therapeutic Target for Human Solid Tumors. Proc Natl Acad Sci USA (2012) 109(17):6662–7. doi: 10.1073/pnas.1121623109 PMC3340046 22451913

[39] Zhang H Lu H Xiang L Bullen JW Zhang C Samanta D. Hif-1 Regulates Cd47 Expression in Breast Cancer Cells to Promote Evasion of Phagocytosis and Maintenance of Cancer Stem Cells. Proc Natl Acad Sci USA (2015) 112(45):E6215–23. doi: 10.1073/pnas.1520032112 PMC4653179 26512116

[40] Michaels AD Newhook TE Adair SJ Morioka S Goudreau BJ Nagdas S. Cd47 Blockade as an Adjuvant Immunotherapy for Resectable Pancreatic Cancer. Clin Cancer Res (2018) 24(6):1415–25. doi: 10.1158/1078-0432.CCR-17-2283 PMC6296745 29288236

[41] Soto-Pantoja DR Terabe M Ghosh A Ridnour LA DeGraff WG Wink DA. Cd47 in the Tumor Microenvironment Limits Cooperation Between Antitumor T-Cell Immunity and Radiotherapy. Cancer Res (2014) 74(23):6771–83. doi: 10.1158/0008-5472.CAN-14-0037-T PMC4253868 25297630

[42] Noman MZ Janji B Berchem G Mami-Chouaib F Chouaib S. Hypoxia-Induced Autophagy: A New Player in Cancer Immunotherapy? Autophagy (2012) 8(4):704–6. doi: 10.4161/auto.19572 22441015

[43] Hasmim M Janji B Khaled M Noman MZ Louache F Bordereaux D. Cutting Edge: Nanog Activates Autophagy Under Hypoxic Stress by Binding to Bnip3l Promoter. J Immunol (2017) 198(4):1423–8. doi: 10.4049/jimmunol.1600981 28093523

[44] Hasmim M Noman MZ Messai Y Bordereaux D Gros G Baud V. Cutting Edge: Hypoxia-Induced Nanog Favors the Intratumoral Infiltration of Regulatory T Cells and Macrophages *Via* Direct Regulation of Tgf-Beta1. J Immunol (2013) 191(12):5802–6. doi: 10.4049/jimmunol.1302140 24227785

[45] Noman MZ Buart S Romero P Ketari S Janji B Mari B. Hypoxia-Inducible Mir-210 Regulates the Susceptibility of Tumor Cells to Lysis by Cytotoxic T Cells. Cancer Res (2012) 72(18):4629–41. doi: 10.1158/0008-5472.CAN-12-1383 22962263

[46] Barsoum IB Hamilton TK Li X Cotechini T Miles EA Siemens DR. Hypoxia Induces Escape From Innate Immunity in Cancer Cells *Via* Increased Expression of Adam10: Role of Nitric Oxide. Cancer Res (2011) 71(24):7433–41. doi: 10.1158/0008-5472.CAN-11-2104 22006996

[47] Yamada N Yamanegi K Ohyama H Hata M Nakasho K Futani H. Hypoxia Downregulates the Expression of Cell Surface Mica Without Increasing Soluble Mica in Osteosarcoma Cells in a Hif-1alpha-Dependent Manner. Int J Oncol (2012) 41(6):2005–12. doi: 10.3892/ijo.2012.1630 22992985

[48] Baginska J Viry E Berchem G Poli A Noman MZ van Moer K. Granzyme B Degradation by Autophagy Decreases Tumor Cell Susceptibility to Natural Killer-Mediated Lysis Under Hypoxia. Proc Natl Acad Sci USA (2013) 110(43):17450–5. doi: 10.1073/pnas.1304790110 PMC3808626 24101526

[49] Messai Y Noman MZ Hasmim M Janji B Tittarelli A Boutet M. Itpr1 Protects Renal Cancer Cells Against Natural Killer Cells by Inducing Autophagy. Cancer Res (2014) 74(23):6820–32. doi: 10.1158/0008-5472.CAN-14-0303 25297632

[50] Sitkovsky M Lukashev D. Regulation of Immune Cells by Local-Tissue Oxygen Tension: Hif1 Alpha and Adenosine Receptors. Nat Rev Immunol (2005) 5(9):712–21. doi: 10.1038/nri1685 16110315

[51] Sitkovsky MV Hatfield S Abbott R Belikoff B Lukashev D Ohta A. Hostile, Hypoxia-A2-Adenosinergic Tumor Biology as the Next Barrier to Overcome for Tumor Immunologists. Cancer Immunol Res (2014) 2(7):598–605. doi: 10.1158/2326-6066.CIR-14-0075 24990240 PMC4331061

[52] Terry S Engelsen AST Buart S Elsayed WS Venkatesh GH Chouaib S. Hypoxia-Driven Intratumor Heterogeneity and Immune Evasion. Cancer Lett (2020) 492:1–10. doi: 10.1016/j.canlet.2020.07.004 32712233

[53] Wigerup C Pahlman S Bexell D. Therapeutic Targeting of Hypoxia and Hypoxia-Inducible Factors in Cancer. Pharmacol Ther (2016) 164:152–69. doi: 10.1016/j.pharmthera.2016.04.009 27139518

[54] Chan MC Holt-Martyn JP Schofield CJ Ratcliffe PJ. Pharmacological Targeting of the Hif Hydroxylases–a New Field in Medicine Development. Mol Aspects Med (2016) 47-48:54–75. doi: 10.1016/j.mam.2016.01.001 26791432

[55] Haase VH. Therapeutic Targeting of the Hif Oxygen-Sensing Pathway: Lessons Learned From Clinical Studies. Exp Cell Res (2017) 356(2):160–5. doi: 10.1016/j.yexcr.2017.05.004 PMC5507591 28483447

